# Hypertension-Mediated Organ Damage in Relation to Severity of Chronic Low Back Pain in Hypertensive Patients

**DOI:** 10.3390/jcdd11090266

**Published:** 2024-08-28

**Authors:** Maciej Skrzypek, Rafał Kolec, Michał Słaboszewski, Katarzyna Góra, Agnieszka Olszanecka, Piotr Wróbel, Katarzyna Stolarz-Skrzypek, Marek W. Rajzer

**Affiliations:** 1 Department of Medicine and Health Sciences, Andrzej Frycz Modrzewski Krakow University, 30-705 Kraków, Poland; 2 Students Scientific Group, First Department of Cardiology, Interventional Electrocardiology and Hypertension, Faculty of Medicine, Jagiellonian University Medical College, 31-008 Kraków, Poland; 3 First Department of Cardiology, Interventional Electrocardiology and Hypertension, Jagiellonian University Medical College, Jakubowskiego 2, 30-688 Kraków, Poland

**Keywords:** Oswestry Disability Index, chronic low back pain, carotid artery plaques, hypertension, organ damage

## Abstract

Introduction: Chronic pain triggers a stress response, which results in increased blood pressure (BP). We investigated whether chronic low back pain (cLBP) in hypertensive patients is associated with an increased risk of hypertension-related organ damage. Methods: We studied 85 consecutive hypertensive patients with a median age of 62 years (55–67), who suffered from cLBP, the severity of which was evaluated according to the Oswestry Disability Index (ODI). Patients underwent transthoracic echocardiography, arterial ultrasonography and vascular tonometry. We assessed carotid artery atherosclerotic plaques, along with carotid-femoral pulse wave velocity (cf-PWV) and left ventricular mass index (LVMI). Results: An equal to or higher than median (16 points) ODI score in 48 subjects (56.5%) was associated with the presence of carotid artery plaques (*p* = 0.014). In multivariate analysis, after adjusting for covariates, the presence of carotid artery plaques remained independently associated with an ODI score equal to or higher than the median (OR, 3.71; 95% CI, 1.04–13.25; *p* = 0.044). None of the other analyzed parameters of hypertension-related organ damage demonstrated a significant relationship with the ODI score. Conclusions: We observed that more severe cLBP is associated with a higher prevalence of carotid artery atherosclerotic plaques among hypertensive patients.

## 1. Introduction

The prevalence of hypertension is growing and it is estimated to increase to a total of 1.56 billion cases by 2025, being one of a leading risk factors for mortality worldwide [1]. Elevated blood pressure (BP) leads to a number of organ complications such as arterial stiffness, retinopathy, left ventricular hypertrophy or chronic kidney disease, which may remain asymptomatic. Hypertension often occurs with hypercholesterolemia, type 2 diabetes, gout and obesity, which form clinically important risk factors for cardiovascular disease [2]. Not only hypertension, but also chronic pain are highly prevalent in the general and geriatric population [3], and both states frequently coexist in clinical practice [4]. Pain triggers a stress response reflected by the activation of the sympathetic nervous system and the hypothalamic–pituitary–adrenal axis, in which released cortisol and catecholamines increase BP and stimulate carotid and aortic baroreceptors, which in turn activate descending pain inhibitory pathways [5,6]. Contrary to acute pain in which baroreflex system reduces BP, baroreceptors’ response to chronic pain could be less effective due to impairments in the descending pain inhibitory pathways or desensitization, which is manifested as hypertension [7]. The altered interactions between overlapping systems modulating cardiovascular function and pain in patients with chronic pain may lead to increased BP [8], but data on vascular atherosclerosis associated with chronic pain in hypertensive patients are scarce.

The aim of this study was to evaluate the relationship between chronic low back pain (cLBP) in hypertensive patients and the prevalence of asymptomatic cardiac and vascular complications according to pain severity expressed by the Oswestry Disability Index (ODI).

## 2. Methods

### 2.1. Study Population

The study patients were recruited from the cohort of regular outpatients attending the hypertension clinic at the First Department of Cardiology, Interventional Electrocardiology and Hypertension, University Hospital in Krakow from May 2021 to April 2022. This outpatient clinic is the reference center for hypertensive patients from the south-east region of Poland. Patients were eligible for the study if they had documented grade 1 or 2 hypertension according to the ESC guidelines [2], which was diagnosed at least one year prior to the recruitment, whether pharmacologically treated or not, and where ambulatory systolic BP was below 170 mmHg. The exclusion criteria were chronic kidney disease with an estimated glomerular filtration rate (eGFR) of <30 mL/min/1.73 m^2^ at recruitment for the study, heart failure class II or higher according to the New York Heart Association (NYHA) classification [9], persistent atrial fibrillation, active cancer and chronic inflammatory disease. Patients with prior myocardial infarction, coronary interventions and stroke, as well as transient ischemic, were ineligible. The patients were asked at their regular visit about their episodes of low back pain during the 3 weeks before the screening. Of the 134 patients who gave a positive reply, 85 consented to participate in the current study. All the patients received antihypertensive medications in the program of combination therapy and none of them was on chronic painkillers. The predominant cause of cLBP was lumbar or lumbosacral discopathy. All patients signed informed consent forms. The study was conducted according to the Declaration of Helsinki and approved by the local Bioethics Committee.

### 2.2. Echocardiography

Echocardiography was performed by an experienced physician using the Vivid E95 device (GE Ultrasound, Horten, Norway). The examinations were performed according to the standards of the Echocardiography Section of the Polish Cardiac Society [10] and further analyzed using an EchoPack v204 workstation integrated with the ViewPoint 6 system (ViewPoint, GE Medical Systems, Horten, Norway). Left ventricular morphology was evaluated using M-Mode and 2-D projections, and the following parameters were analyzed: left ventricular systolic diameter (LVSD) and left ventricular diastolic diameter (LVDD), interventricular septum systolic diameter (IVSs), interventricular septum diastolic diameter (IVSd), posterior wall systolic diameter (PWs) and posterior wall diastolic diameter (PWd). The left ventricular ejection fraction (LVEF) was calculated with the use of the Simpson’s biplane method. Left ventricular mass (LVM) was calculated using the Devereux cube formula as follows:

(0.8 × [1.04 (IVSd + LVDD + PWd)^3^ − (LVDD)^3^] + 0.6 g)

and then indexed to the body surface area (BSA) to obtain the left ventricular mass index (LVMI). The biplane disk summation technique was used to obtain left atrial volume (LAV), which was indexed to BSA to achieve left atrial volume index (LAVI). The left atrial diameter was estimated by 2-D parasternal long axis view (PLAX) projection measurement.

### 2.3. Carotid Ultrasonography

Intima-media thickness (IMT) complex was measured on both common carotid arteries using the General Electric Vivid E95 device (GE Ultrasound, Horten, Norway) with a linear probe 4.5–12 MHz (GE 11L). IMT was assessed using a semi-automatic method with EchoPAC PC v204 Software Only (GE Medical Systems, Horten, Norway). It involves tracing of 1 cm (starting approximately 1 cm proximally from the bifurcation) of the leading edge of the intima surface and the leading edge of the adventitia surface, followed by multiple measurements between pairs of pixels located on both traces [11]. Carotid IMT parameter describes the IMT presented as the mean average of left and right carotid artery measurements. Carotid atherosclerotic plaques were defined as the presence of IMT > 1.5 mm on either side or thickening of the intima-media complex more than 50% relative to the adjacent arterial wall.

### 2.4. Aortic Pulse Wave Velocity Examination

Prior to the examination, patients rested for 15 min in the supine position. Measurement of the aortic pulse wave velocity (PWV) was calculated from sequential recordings of the pulse wave on the carotid and femoral artery (cf-PWV, carotid to femoral pulse wave velocity) with a simultaneous electrocardiogram. Measurements were made with a Micro-Tip pressure transducer Model SPT-301 (Millar Instruments, Houston, TX, USA) built into the probe and paired with a portable computer equipped with SphygmoCor software, version 6.31 (AtCor Medical Pty., Ltd., Sydney, Australia). Each patient underwent an 8 s tonometric pulse wave measurement at both locations. PWV was calculated as the ratio of distance (meters) to time (seconds).

### 2.5. ODI Questionnaire

The patients independently filled out an ODI questionnaire that included questions on cLBP intensity and variability over time, lifting objects, sitting, sleeping, traveling, grooming, walking, standing, social life and changes in pain intensity. Each response was scored on a scale from 0 to 5, and the maximum number of points scored in the entire questionnaire can reach 50. A higher score was associated with an increased degree of disability.

### 2.6. Other Measurements

A standardized questionnaire was conducted to obtain information on the medical history and habits of individual patients. Current smoking was defined as daily use of tobacco products. Body mass index (BMI) was defined as the patient’s weight, given in kilograms, divided by the square height, given in meters. Diabetes was identified when fasting blood glucose was higher than 7.0 mmol/L (126 mg/dL) on two separate occasions, when glycated hemoglobin was above 6.5% or when there was a previous diagnosis [12]. Hyperlipidemia was coded when total cholesterol levels exceeded 5.0 mmol/L or low-density lipoprotein cholesterol (LDL-C) levels exceeded 3.0 mmol/L or when there was a previous diagnosis. Lipid-lowering therapy was coded if the patient was taking a statin or fibrate treatment. The patients were instructed to remain fasting for at least 8 h before blood collection. Blood for the laboratory tests was collected between 8 and 11 a.m. from an antecubital vein with minimal stasis. The contents of the tubes were then mixed with 3.2% sodium citrate at a ratio of 9:1, followed by centrifugation (20 min, 1500× *g*). Glucose, lipid profile, serum creatinine and eGFR were assessed by standard automated techniques. Ambulatory blood pressure monitoring (ABPM) was performed to assess the average values of systolic BP, diastolic BP and mean heart rate during the day. Measurements were taken at 15 min intervals during the day (from 6 a.m. to 10 p.m.) and every 30 min during the night (from 10 p.m. to 6 a.m.). Prior to the start of the measurements, patients were instructed on their behavior during the study (with suggestions of performing daily activities except for intense exercise, adopting a position with a motionless arm at heart level while taking measurements and keeping a diary of accompanying symptoms during the study period).

### 2.7. Statistical Analysis

The Shapiro–Wilk test was used to verify the normality of the distribution of each variable. The continuous variables were presented as medians (interquartile range) and as means (standard deviation) for non-normal and normal distributions, respectively. The Mann–Whitney U test or Student’s *t*-test were used to compare the continuous variables, as applicable. The categoric variables were presented as numbers (percentages) and compared by Pearson’s χ^2^ test or Fisher’s exact test. Multivariate logistic analysis was used to identify the factors associated with equal to or higher than median ODI scores. The results of the logistic regression models were presented on a forest plot, using odds ratios (ORs) with 95% confidence intervals (CIs). Statistical analyses were performed using STATISTICA 13.0 (2017; TIBCO Software Inc., Palo Alto, CA, USA). The level of statistical significance was defined as *p* < 0.05.

## 3. Results

### 3.1. Patient Characteristics

The study population included 85 patients with an average age of 62 years (55–67), of whom 56 (65.88%) were women and 29 (34.12%) were men. The average BMI of the cohort was 28.98 (±4.34) kg/m^2^. Males were similar to females in terms of demographic and clinical variables with the exception of 24 h diastolic BP and serum creatinine concentration, which were higher in men (*p* = 0.032; *p* < 0.001, respectively), as shown in Table 1.

### 3.2. Baseline Echocardiographic and Vascular Characteristics

As expected, the men differed from the women in terms of echocardiographic parameters, as they had a larger LVDD (*p* = 0.008), as well as a greater IVSs and IVSd (both *p* = 0.001). In the male group, the PWs and PWd were thicker (*p* = 0.002; *p* = 0.001, respectively), as compared to the females. The men had a higher LVM (*p* = 0.011), which resulted in a higher LVMI (*p* = 0.011) compared with the remainder. The left atrium diameter was larger in the males as compared to the females (*p* = 0.009). The data are shown in Table 2.

### 3.3. ODI Score and Cardiac or Vascular Characteristics

In the whole study population, the median ODI questionnaire score was 16, while the interquartile range was from 11 to 20. The results of the univariate analysis on the relationship between the cardiac and vascular parameters in the patients divided into two groups (1—ODI score lower than median or 2—ODI score equal/higher than median) showed that in subjects with a higher disability score, carotid artery plaques were diagnosed more often as compared with the remainder (*p* = 0.014). There were no significant differences between the groups in terms of other cardiac and vascular parameters. The results are shown in Table 3.

### 3.4. Regression Analysis

**Figure 1:**
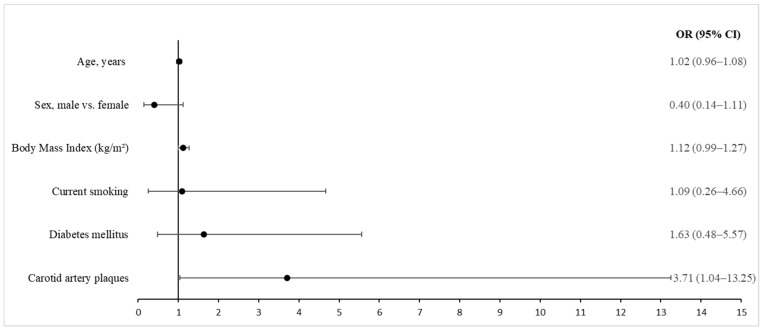
Factors associated with equal to or higher than median Oswestry Disability Index score in the multivariable regression model.

To adjust for confounding factors, we performed a multivariable regression analysis on the association between the presence of carotid artery plaques and equal to or higher than median ODI scores, while accounting for other variables (Figure 1). The final model included age, gender, BMI, smoking and diabetes mellitus. With adjustment for covariates, we confirmed a significant relationship between the presence of carotid artery plaques and equal to or higher than median ODI scores (OR, 3.71; 95% CI, 1.04–13.25; *p* = 0.044). We also performed a multivariable regression model with similar covariates for LVM, LVMI, LAVI and carotid IMT, but none of these models provided significant results.

## 4. Discussion

To our knowledge, this study is the first to demonstrate an association between the occurrence of carotid artery atherosclerotic plaques in hypertensive patients and cLBP reflected by a higher ODI score.

The study group was representative of typical elderly patients with hypertension in terms of demographic and clinical characteristics [13]. Consistent with previous papers, serum creatinine levels were higher in the men as compared with the women [14]. Nonsteroidal Anti-Inflammatory Drugs (NSAIDs) along with acetaminophen are known to increase BP [15]; however, our patients were not routinely subjected to such pharmacotherapy.

Prior reports on BP trends with respect to gender have presented results consistent with our observations shown in Table 1. According to the above, systolic BP values among the males and females did not differ significantly at the median age of ~60 years. However, diastolic BP values for the same median age were significantly higher in the men than in the women [16].

The difference in the echocardiographic measurements of the heart chambers and walls between the males and females in our study population was expected, as this were validated for gender following the recommendations of the guidelines on echocardiographic quantification [17].

In a study of 23,632 Koreans, a logistic regression analysis adjusted for age has shown an association between the presence of cLBP and cardiovascular or cerebrovascular diseases (cardiac infarction, angina, ischemic stroke) [18]. A systematic review on the relationship between atherosclerosis and cLBP, analyzing 26 original studies, has shown an association between carotid artery IMT and the occurrence of sciatica, but not cLBP. Moreover, this association was reported only in men [19]. Our study expands the previous findings on vascular complications in patients with cLBP by showing that carotid artery atherosclerosis defined as the presence of carotid artery plaques is associated with more severe cLBP co-existing with arterial hypertension. In our study, we did not examine vascular beds other than the aforementioned carotid arteries, as the assessment of coronary plaques is not included in the routine screening of hypertensive patients for organ damage. None of the patients recruited for the study presented any clinical signs of myocardial ischemia.

Previous studies have shown a relationship between the presence of hypertension and bothersome or activity-limiting pain in various locations, including the back. Contrary to our study, the above study did not use pain assessment questionnaires, and patients were included in the pain-reporting group based on the presence of pain, its localization and severity [20]. According to the risk factors associated with vascular damage in patients with atherosclerosis, we can distinguish dyslipidemia, diabetes mellitus, obesity or the aforementioned hypertension, which not only induces but also is responsible for the development and progression of the atherogenesis process [21]. Atherosclerotic plaques found inside the carotid arteries may be an expression of the next stage of atherogenesis associated with inflammation, oxidative stress, endothelial dysfunction or smooth muscle cell proliferation [22]. One of the previously mentioned risk factors for cardiovascular disease by promoting atherogenesis is dyslipidemia [23]. Its impact could have interfered with the study by affecting the incidence of carotid artery plaques. However, in both groups with ODI scores equal to/higher than 16 and below 16, the number of subjects with poorly controlled lipid disorder was equal. Carotid atherosclerosis and its related parameters, such as the presence of carotid artery plaques or carotid IMT, were analyzed in prior studies regarding their predictive value for the occurrence of cardiac diseases. Inaba et al. pooled results from a population-based study and showed that the presence of carotid artery plaques had a higher diagnostic value in predicting future myocardial infarction and coronary artery disease than did carotid IMT [24]. We hypothesize that the lack of difference in carotid IMT in our cohort with regard to the severity of cLBP may be related to the fact that only seven (8.24%) individuals have carotid IMT of more than 0.9 mm, which is considered to be abnormal, and current guidelines still point out for the need for further investigations to establish the value of carotid IMT in cardiovascular risk stratification [25].

Based on our results, we would suggest that in-depth medical interviews with hypertensive patients should be focused also on the occurrence of cLBP among these patients, which may help decide whether the diagnostics should be expanded to include ultrasonography of carotid arteries as well as other vascular beds. If atherosclerotic plaques are detected, this finding might assign the patient to a higher cardiovascular risk category aiming at the prescription of lipid-lowering treatment to reduce the risk of future events [25,26]. However, despite strong guidelines supporting the use of statins, worries about side effects, especially musculoskeletal symptoms, contribute to statin intolerance and patient reluctance to continue therapy [27].

Further studies are needed to determine the effect of cLBP on the incidence of atherosclerosis in the coronary arteries and other vascular beds such as lower extremity arteries. Moreover, some clarification is also indispensable regarding the aspect of administering prophylactic doses of antiplatelet drugs in a group of patients with carotid atherosclerotic plaques. Additionally, it is necessary to confirm the observations presented in our study on a larger subject population.

## 5. Study Limitations

This study has several limitations. First, the sample size is relatively small, but sufficient to detect differences in vascular parameters between the groups. Second, it was conducted in a single center; however, this center serves a large population of patients. The outpatients were examined by a certified investigator with long-standing experience, who conducted both the vascular ultrasonography and echocardiography. Finally, the ODI questionnaire may not be sensitive enough to detect subtle differences, especially in patients with very mild or very severe disability, which may affect their final score. It does not fully contain questions on the emotional, social and psychological aspects of a patient’s life, which may remain important to assess pain severity. However, a study comparing five commonly used questionnaires assessing disability among patients with cLBP found that ODI has a sufficient reliability and scale range to be used in an outpatient clinical population with cLBP [28]. As a test, the internal consistency according to the Cronbach’s α for the ODI questionnaire ranges from 0.71–0.87 and has high test–retest reliability (intraclass correlation coefficient is 0.84; 95% CI, 0.73–0.91). The standard error of measurement ranges from four to six [29]. The ODI is one of the better-known tests used to measure disability degree in cLBP patients—not only in clinical practice, but also in research [30,31]. In our study, we did not detect any association between the severity of cLBP and the echocardiographic parameters. However, speckle tracking echocardiography, an innovative imaging method for the assessment of subclinical myocardial dysfunction [32], was not performed in the present study. Finally, whether cLBP has a causal role in carotid atherosclerotic plaque formation was beyond the scope of this study.

## 6. Conclusions

To conclude, we observed that more severe cLBP is associated with a higher prevalence of carotid artery atherosclerotic plaques among hypertensive patients, which should influence the hypertension management of this subgroup of patients.

## Figures and Tables

**Table 1:** Baseline patient characteristics by gender.

Variable	Male (*n* = 29)	Female (*n* = 56)	*p* Value
Median age (years)	61.00 (57.00–67.00)	63.00 (54.00–66.00)	0.91
BMI (kg/m^2^)	28.76 ± 3.37	29.09 ± 4.79	0.74
Systolic blood pressure 24 h (mm Hg)	121.00 (114.00–136.00)	118.50 (113.50–126.50)	0.15
Diastolic blood pressure 24 h (mm Hg)	73.00 (69.00–85.00)	70.00 (66.00–77.50)	**0.032**
Heart rate 24 h (beats/min)	69.00 (61.00–74.00)	68.00 (63.00–75.00)	0.54
Hyperlipidemia, n (%)	24 (82.76)	47 (83.93)	0.87
Lipid-lowering drugs, n (%)	23 (79.31)	37 (66.07)	0.31
Diabetes mellitus, n (%)	9 (31.03)	14 (25.00)	0.74
Current smoking, n (%)	2 (6.90)	9 (16.07)	0.32
Serum creatinine (μmol/L)	86.60 (76.00–93.00)	69.50 (62.70–78.10)	**<0.001**
eGFR (mL/min/1.73 m^2^)	81.00 (74.00–90.00)	78.00 (69.00–90.00)	0.30
Serum LDL-C, (mmol/L)	2.10 (1.90–2.65)	2.50 (1.85–3.37)	0.21

Abbreviations: BMI—body mass index; eGFR—estimated glomerular filtration rate; LDL-C—low-density lipoprotein cholesterol. The statistical significance at which the data were bolded was 0.05.

**Table 2:** Baseline echocardiographic and vascular parameters by gender.

Variable	Male (*n* = 29)	Female (*n* = 56)	*p* Value
LVSD (mm)	30.17 ± 3.95	29.07 ± 4.11	0.24
LVDD (mm)	49.62 ± 4.98	46.64 ± 4.73	**0.008**
IVSs (mm)	18.00 (16.00–20.00)	16.00 (15.00–17.00)	**<0.001**
IVSd (mm)	11.00 (11.00–12.00)	10.00 (9.50–11.50)	**<0.001**
PWs (mm)	17.00 (16.00–17.00)	15.00 (14.00–16.00)	**0.002**
PWd (mm)	11.00 (10.00–12.00)	10.00 (9.00–11.00)	**0.001**
LVEF (%)	70.00 (65.00–74.00)	65.50 (60.50–73.00)	0.19
LVM (g)	214.25 ± 43.44	170.07 ± 41.17	**<0.001**
LVMI (g/m^2^)	105.43 ± 20.40	93.69 ± 19.33	**0.011**
LA diameter (mm)	39.48 ± 4.45	36.93 ± 4.02	**0.009**
LAVI (mL/m^2^)	30.07 ± 9.36	33.18 ± 8.96	0.14
Carotid IMT (mm)	0.60 (0.50–0.70)	0.60 (0.55–0.70)	0.58
Carotid artery plaques, n (%)	9 (31.03)	15 (26.79)	0.87
PWV (m/s)	8.60 (7.70–9.90)	8.05 (7.20–9.25)	0.24

Abbreviations: IVSd—interventricular septum diastolic diameter; IVSs—interventricular septum systolic diameter; IMT—intima-media thickness; LAVI—left atrial volume index; LA—left atrium; LVDD—left ventricular diastolic diameter; LVEF—left ventricular ejection fraction; LVM—left ventricular mass; LVMI—left ventricular mass index; LVSD—left ventricular systolic diameter; PWd—posterior wall diastolic diameter; PWs—posterior wall systolic diameter; PWV—pulse-wave velocity. The statistical significance at which the data were bolded was 0.05.

**Table 3:** Results of univariate analysis on the relationship between cardiac or vascular parameters and ODI score.

Variable	Oswestry Disability Index		*p* Value
	<16	≥16	
LVMI (g/m^2^)	95.56 ± 18.69	99.35 ± 21.62	0.4
LAVI (mL/m^2^)	31.01 ± 9.15	32.98 ± 9.18	0.33
Carotid IMT (mm)	0.6 (0.55—0.75)	0.6 (0.53–0.70)	0.96
Carotid artery plaques, n (%)	5 (13.51)	19 (39.58)	**0.014**
PWV (m/s)	8.05 (7.30–9.50)	8.60 (7.20–9.50)	0.9

Abbreviations: IMT—intima-media thickness; LAVI—left atrial volume index; LVMI—left ventricular mass index; PWV—pulse-wave velocity. The statistical significance at which the data were bolded was 0.05.

## Data Availability

The data presented in this study are available on request from the corresponding author due to legal restrictions, i.e., personal and medical data protection law.
